# Synthesis of silver nanostructure on gold nanoparticle using near field assisted second harmonic generation

**DOI:** 10.1038/s41598-021-84944-w

**Published:** 2021-03-11

**Authors:** Takashi Yatsui, Felix Brandenburg, Benjamin Leuschel, Olivier Soppera

**Affiliations:** 1 Toyohashi University of Technology, 1-1 Hibarigaoka, Tenpaku-cho, Toyohashi, Aichi 441-8580 Japan; 2 The University of Tokyo, 7-3-1 Hongo, Bunkyo-ku, Tokyo 113-8656 Japan; 3 Université de Haute-Alsace, CNRS, IS2M UMR 7361, 68100 Mulhouse, France; 4 Université de Strasbourg, Strasbourg, France

**Keywords:** Nanoparticles, Nanophotonics and plasmonics

## Abstract

By using gold (Au) nanoparticles (NPs) as an optical near-field source under far-field illumination in combination with a silver (Ag) ion solution containing a photoinitiator, we coated Ag on Au NPs using a near-field (NF)-assisted process. We evaluated the change in the size of the NPs using transmission electron microscopy. Evaluation of the synthesized Ag volume over illumination power confirmed the squared power dependence of the NP volume with illumination using 808 nm light, i.e., a wavelength longer than the absorption edge wavelength of the photoinitiator molecules. The rate of volume increase was much lower than the plasmonic field enhancement effect. Therefore, the squared power dependency of the volume increase using a wavelength longer than the absorption edge wavelength originated from NF-assisted second-harmonic generation and the resulting excitation.

## Introduction

Photochemical reactions have been widely used to enhance various chemical transformations involving H_2_ generation^1^, CO_2_ reduction^2^, isomerization^3^, and polymerization of coatings and 3D printing materials^4^. Because photochemical reactions are based on the photoexcitation of molecules, the wavelength employed for the photochemical reaction should be shorter than the highest occupied molecular orbital (HOMO)-lowest unoccupied molecular orbital (LUMO) gap (HLG) wavelength. However, it is possible to utilize a wavelength longer than the HLG wavelength, but this requires intense light illumination for high harmonic generation^5^. As an alternative approach, we have developed an approach that utilizes the unique properties of an optical near-field (ONF) source^6^. As the ONF is localized in the nanoscale range, it therefore has a non-uniform optical field in the nanoscale range, meaning that the field gradient $\left( {\frac{\partial E}{{\partial r}}} \right)$ is not zero for nanoscale materials. In such a system, the ONF inherently achieves strong second-harmonic generation (SHG) without intense light illumination because the ONF breaks the selection rules of SHG^7,8^. It is noted that the ONF-assisted SHG does not require the strong optical field which was achieved by the intense light illumination in the conventional approach^5^. Furthermore, the ONF can excite dipole forbidden transitions of various molecules^6^ via intermediate states inside the HLG, making it possible to utilize wavelengths longer than the HLG wavelength. By harnessing such properties of the ONF, we have improved the efficiency of photochemical reactions, including those involved in H_2_ generation^9^, CO_2_ reduction^10,11^, photolithography^12^, and photodimerization^13^ using wavelengths longer than the HLG wavelength, which expands the possibilities for excitation of resonant transitions demonstrated in other works^14–16^. Although it is possible to utilize a wavelength longer than the HLG wavelength, phenomena associated with far-field (FF) properties were observed—such as changes in the photocurrent^9^ or absorption spectra^10,11,13^—and to date there remains no direct proof of the ONF effect. Herein, transmission electron microscopy (TEM) is employed to obtain direct proof of the ONF-based reaction by measuring the change in size of nanostructures at the atomic level.

### Near-field-assisted silver synthesis on gold nanoparticles

**Figure 1:**
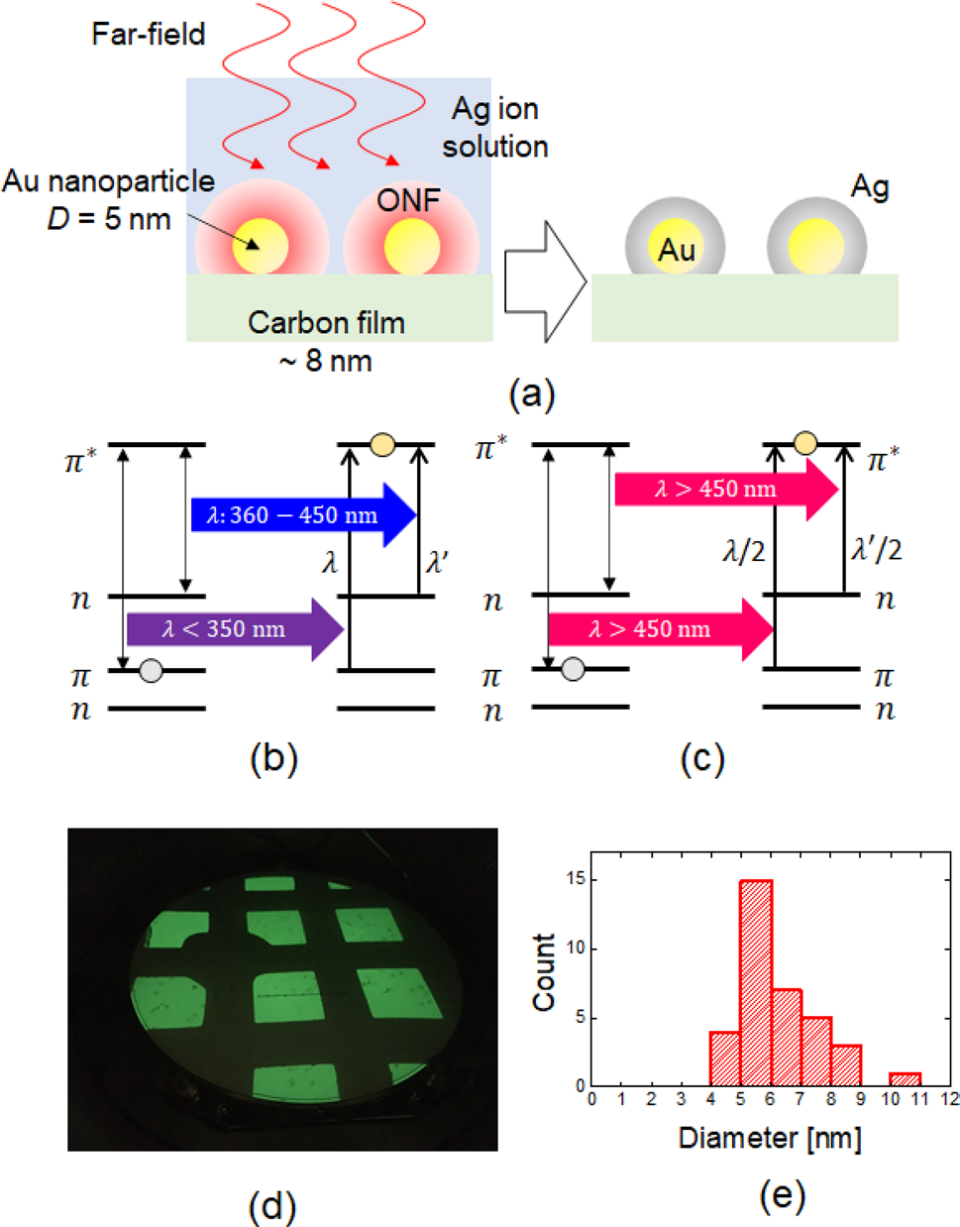
(**a**) Schematic diagram showing the process of near-field assisted Ag deposition on Au NPs. The optical near-field (ONF) is generated on the Au NPs upon irradiation with far-field light. Energy transition diagrams illustrating (**b**) FF and (**c**) NF excitations of $\pi - \pi^{*}$ and $n - \pi^{*}$ transitions in the photocleavage of C–P bonds. (**d**) Photograph of the TEM carbon film on the TEM grid. (**e**) Size distribution of the Au NPs before irradiation.

Figure 1a schematically shows the near-field (NF)-assisted process of Ag deposition on Au nanoparticles (NPs). Au NPs (5 nm diameter) were used as the ONF source. We chose a different material to Au for the core material, thus allowing us to recognize the size growth that can be realized through the deposition process. These Au NPs were immersed in a photosensitive Ag ion aqueous solution containing 0.1 wt% AgNO_3_ and 0.2 wt% Irgacure 819 as an initiator^17^. The Ag ions dissolved in water can be reduced to form neutrally charged agglomerates upon irradiation at wavelengths shorter than the absorption edge wavelength (*λ*_edge_; i.e., 450 nm) according to the following equations.12

It is evident from Eq. (1) that, in the presence of Irgacure 819, the reduction of Ag^+^ to Ag^0^ and the Ag deposition involve both benzoyl and phosphonyl primary radicals. These radicals result from the cleavage of the C–P bond.

Irgacure 819 exhibits absorption peaks at < 350 nm and 360–450 nm^17^, corresponding to $\pi - \pi^{*}$ and $n - \pi^{*}$ transitions^18^, respectively (Fig. 1b). Therefore, when the wavelength of incident light from the FF light source is shorter than the *λ*_edge_, silver metal can be produced by direct photoexcitation. Conversely, if the wavelength is longer than the *λ*_edge_, there is no possibility for direct photoexcitation of Irgacure 819. However, it is expected that photoexcitation can occur from a combination of the FF and the NF effects by introducing Au NPs, as the ONFs are induced in the vicinity of the Au NPs, resulting in the excitation by the NF-assisted SHG. Figure 1c shows the NF-assisted SHG and the subsequent excitation of Irgacure 819.

To evaluate Au NPs before and after FF illumination via TEM, Au NPs were deposited on carbon film on the TEM grid, and the size and shape of the same Au NPs were compared (Fig. 1d). In other words, TEM reveals the growth of a silver layer on the Au NP surface, which is used as an indicator and direct proof for the existence and quantity of localized ONFs. The Au NPs chosen were ~ 5 nm (Fig. 1e) and deposition of Ag on the Au NPs was performed using a 325 nm laser (shorter than the *λ*_edge_) as the FF light source and an 808 nm laser (longer than the *λ*_edge_) as the NF light source. Note that the 325 nm laser also generates the ONF on the Au NPs; however, the Ag deposition rate resulting from the FF is significantly higher than that arising from the ONF. Therefore, the Ag deposition of the ONF using the 325 nm laser can be neglected.

## Results and discussion

**Figure 2:**
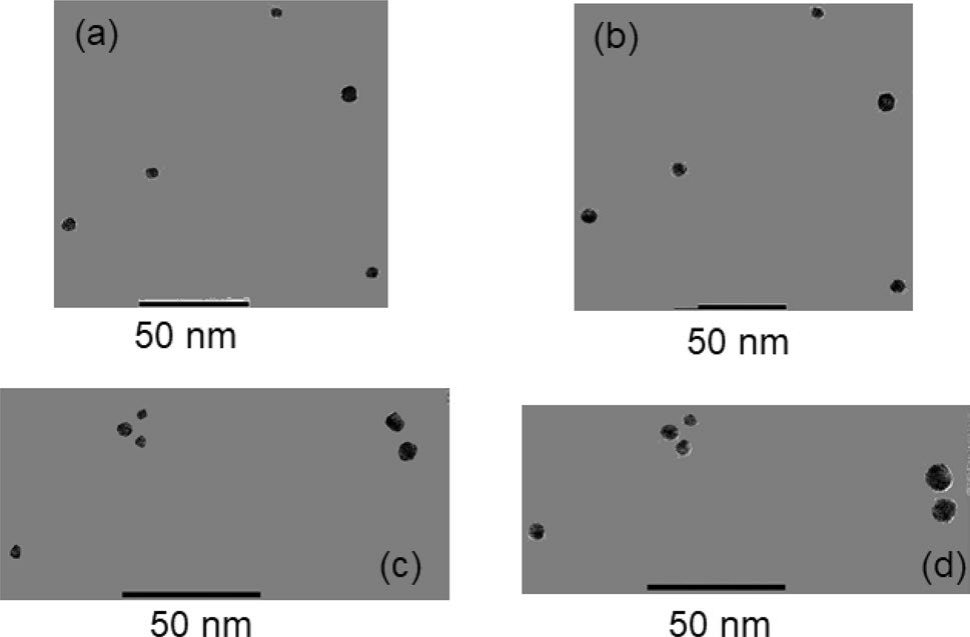
Representative TEM images (**a**) before and (**b**) after irradiation at 325 nm (9 mW for 90 s), and (**c**) before and (**d**) after irradiation at 808 nm (400 mW for 15 min).

**Figure 3:**
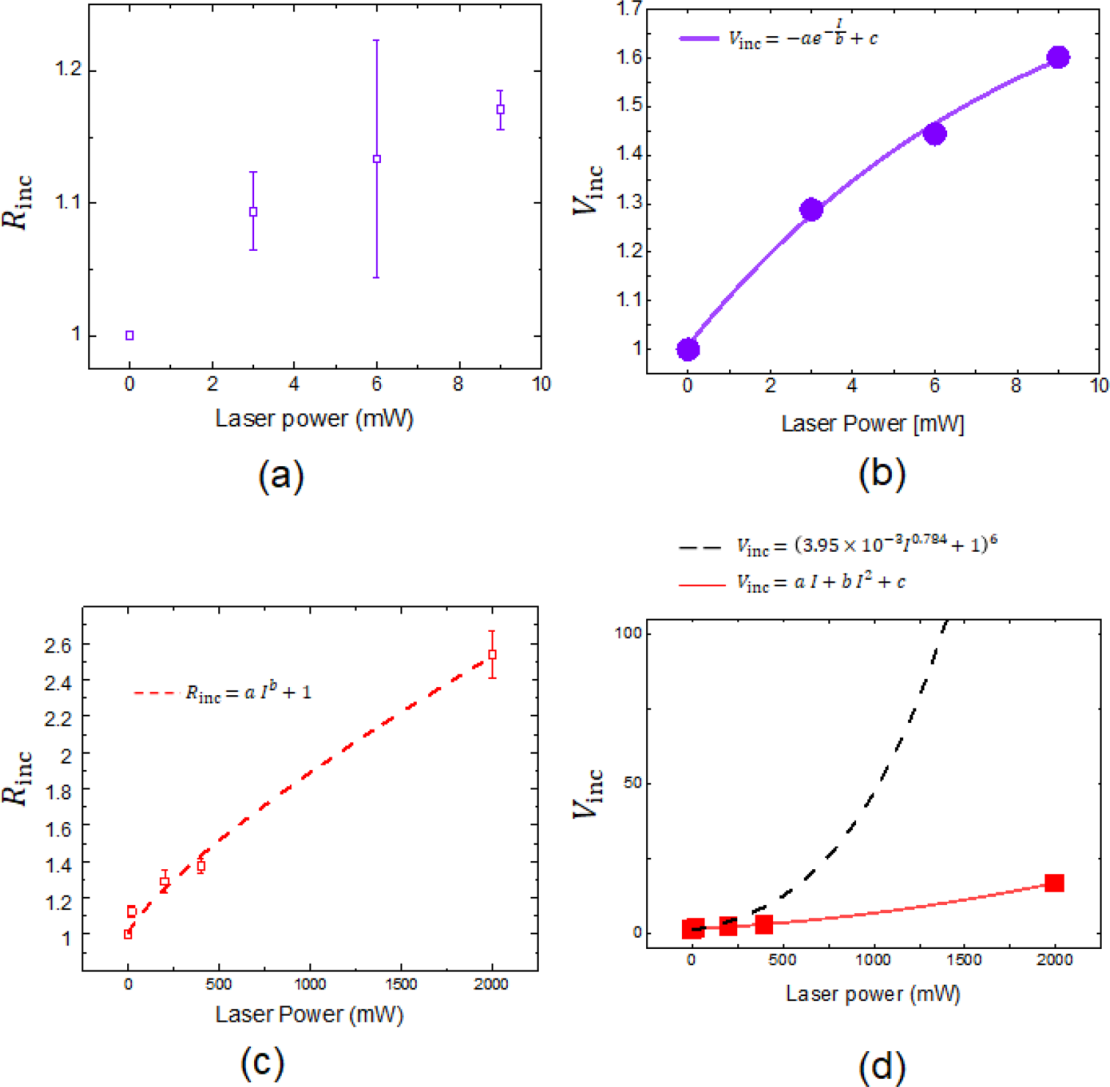
Power and wavelength dependence of Ag deposition on Au NPs. (**a**) Relative radius increase ($R_{{{\text{inc}}}}$) for 325 nm irradiation. (**b**) Volume increase ratio ($V_{{{\text{inc}}}}$ $= \overline{{R_{{{\text{inc}}}} }}^{3}$) for 325 nm irradiation and fitted curve of $V_{{{\text{inc}}}} = a{\text{ exp}}\left( { - I/b} \right) + c$, where $I$ is the incident laser power. (**c**) $R_{{{\text{inc}}}}$ for 808 nm irradiation and fitted curve of $R_{{{\text{inc}}}} = a I^{b} + 1$. (**d**) $V_{{{\text{inc}}}}$ for 808 nm irradiation with fitted solid red curve of $V_{{{\text{inc}}}} = a I + b I^{2} + c$ for the near-field effect and fitted black dashed curve of $V_{{{\text{inc}}}} = \left( {3.95 \times 10^{ - 3} I^{0.787} + 1} \right)^{6}$ for plasmon resonance.

Figure 2 shows representative examples of the TEM images obtained for Au NPs before and after irradiation (all images acquired after irradiation are of the same Au NPs as before irradiation). Note that Ag formed on the Au spheres only after irradiation at 808 nm, as 808 nm is far from the *λ*_edge_, and therefore not enough photon energy is provided to start the photoactivation process by the FF process. Therefore, it is expected that the Ag deposition originated from the ONF process in the presence of the 808 nm laser illumination. The small beaker containing the Irgacure 819 was covered by aluminum foils before and after irradiation; moreover, all the experiments were performed under the dark condition with the room lights turned off. Therefore, the photochemical reactions arising from the room lights were avoided. The Feret diameter^19^ (i.e., the maximum diameter) of each Au NP from the TEM images was first observed. Because the Au NPs vary in size (Fig. 1e), the relative radius increase, $R_{{{\text{inc}}}}$, defined by Eq. (3) was evaluated next.

3 $$R_{{{\text{inc}}}} = \frac{{r_{a} }}{{r_{b} }}$$

where $r_{b}$ and $r_{a}$ are the Feret diameter before and after illumination of the Au NPs (please refer to section S1 in the Supplementary Information for detailed evaluation of the NP size). Figure 3 shows the power and wavelength dependence of the $R_{{{\text{inc}}}}$. The volume increase ratio $\left( {V_{{{\text{inc}}}} \left( { = \overline{{R_{{{\text{inc}}}} }}^{3} } \right)} \right)$, where $\overline{{R_{{{\text{inc}}}} }}$ is the mean $R_{{{\text{inc}}}}$, was then obtained.

Figure 3a shows the power dependence of $R_{{{\text{inc}}}}$ from FF-based excitation at 325 nm. Using the mean $R_{{{\text{inc}}}}$ (Fig. 3a), the corresponding power dependence of $V_{{{\text{inc}}}}$ (Fig. 3b) was obtained. As shown from the fit of $V_{{{\text{inc}}}} = a {\text{exp}}\left( { - I/b} \right) + c$ (fitting parameters shown in Table 1), where $I$ is the incident laser power (Fig. 3b), the power dependence has a saturation power dependency as $I$ increases with an appreciable coefficient of determination (COD) of 0.997. This value is exceedingly close to 1. It is expected that $V_{{{\text{inc}}}}$ is proportional to the number of Ag atoms deposited by the photochemical reaction. This saturation behavior is reasonable for FF excitation because at some point, excess photons do not react with the Irgacure 819 located near the Au NPs. Similarly, evaluation for NF-based excitation with a wavelength of 808 nm was performed. First, the power dependence of $R_{{{\text{inc}}}}$ (Fig. 3c) was obtained. In this result, there were small standard deviations, as indicated by the error bars; therefore, we consider the growth rate dependence on the size to be comparatively less than that on the incident power. Subsequently, the power dependence was fitted with $R_{{{\text{inc}}}} = a \times I^{b} + 1$ (fitting parameters shown in Table 1), following which the power dependence of $V_{{{\text{inc}}}}$ (Fig. 3d) was acquired. The fitting curve of $V_{{{\text{inc}}}} = a \times I + b \times I^{2} + c$ (fitting parameters shown in Table 1) indicates that the $V_{{{\text{inc}}}}$ exhibits $I^{2}$ dependence. As show in Table 1, the COD values of Fig. 3c, d are close to 1, implying that the curves were well fitted with the experimental data. The black dashed curve in Fig. 3d is followed by $V_{{{\text{inc}}}} = \left( {R_{{{\text{inc}}}} } \right)^{6}$ for plasmon resonance, and the details will be discussed later. We consider that the linear term ($a$ in $V_{{{\text{inc}}}} = a I + b I^{2} + c$) originated from the excitation with the original photon energy because there was very weak absorbance at 808 nm, implying that there is a possibility of single-photon excitation with a low photon energy. The absorbance of Irgacure 819 was $2.62 \times 10^{ - 4}$ and 3.35 (a 2.24 $\times 10^{3}$-fold increase) for 808 nm and 404 nm, respectively (see the details in section S3 in the Supplementary Information). From the theoretical study, the excitation and dissociation efficiencies from SHG will be of the order of 10^–6^^8^. Therefore, we expect that the second-order term ($b$ in $V_{{{\text{inc}}}} = a I + b I^{2} + c$) is a factor of approximately $10^{3}$ larger than the first-order term. This is comparable to the obtained ratio $b/a$ = 0.87 $\times 10^{3}$.

**Table 1:** Fitting parameters.

	*a*	SE	*b*	SE	*c*	SE	COD
$V_{{{\text{inc}}}} = a {\text{exp}}\left( { - I/b} \right) + c$ in Fig. 2b	− 0.89	0.20	8.37	3.17	1.90	0.21	0.997
$R_{{{\text{inc}}}} = a I^{b} + 1$ in Fig. 2c	3.95 $\times$ 10^–3^	2.56 $\times$ 10^–3^	7.84 $\times$ 10^–1^	8.71 $\times$ 10^–2^			0.991
$V_{{{\text{inc}}}} = a I + b I^{2} + c$ in Fig. 2d	2.87 $\times$ 10^–3^	1.10 $\times$ 10^–3^	2.46 $\times$ 10^–6^	5.22 $\times$ 10^–7^	1.24	0.20	0.999

*SE* standard error, *COD* coefficient of determination.

**Figure 4:**
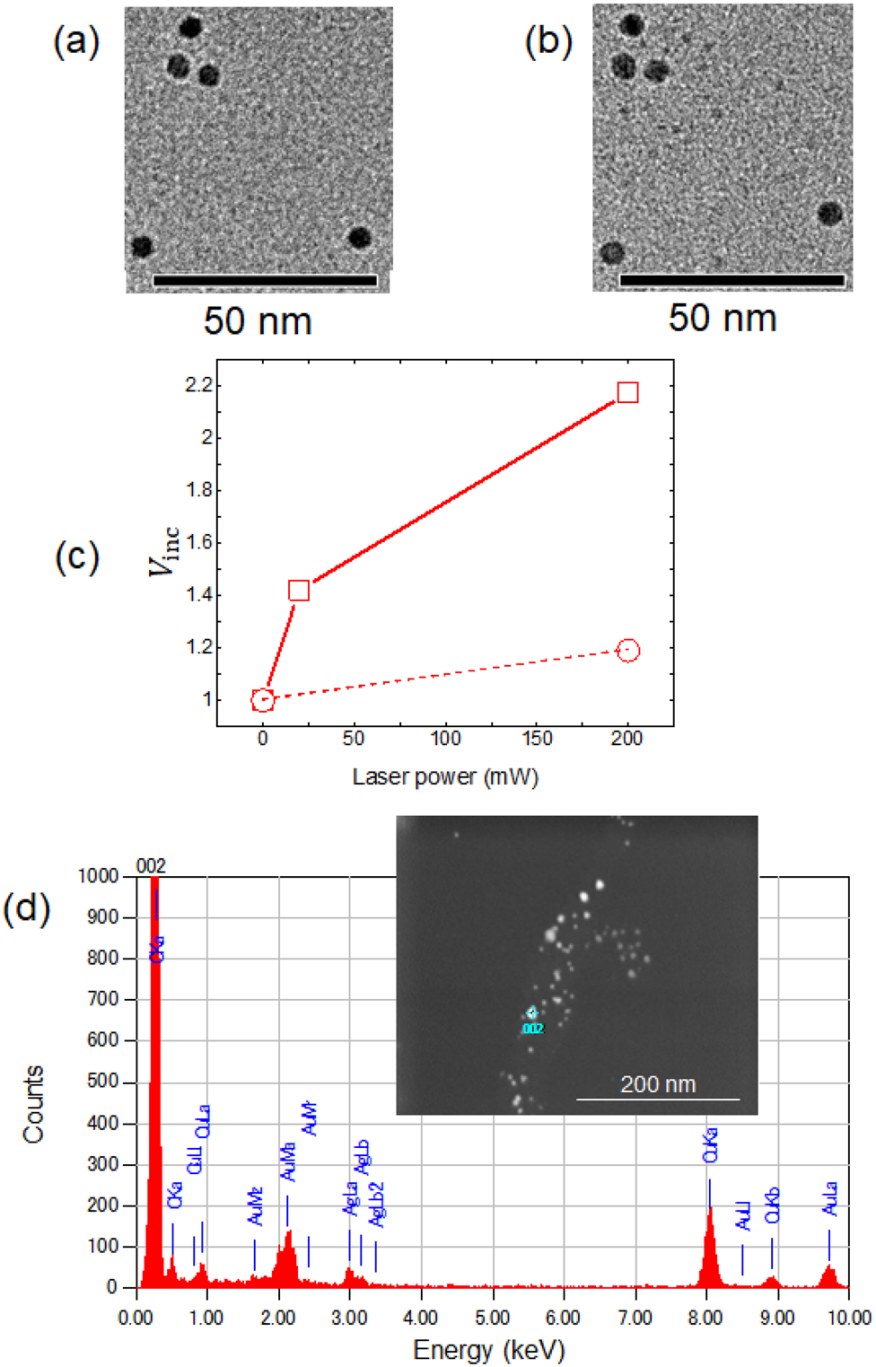
TEM images (**a**) before and (**b**) after 808 nm irradiation (200 mW for 15 min) without photosensitive agent. (**c**) $V_{{{\text{inc}}}}$ for 808 nm (200 mW for 15 min) with Irgacure 819 (squares) and without Irgacure 819 (circles). (**d**) EDS spectrum. Inset: typical TEM image after 808 nm irradiation with Irgacure 819.

To ensure that photochemical Ag deposition was caused by ONFs, 808 nm irradiation (200 mW) was repeated without including the photosensitive agent, Irgacure 819. TEM images (Fig. 4a,b) were obtained in the same manner as described above, and $V_{{{\text{inc}}}}$ was compared with and without Irgacure 819 (Fig. 4c). As shown in Fig. 4c, the $V_{{{\text{inc}}}}$ obtained without Irgacure 819 (open squares) remained the same after 808 nm irradiation for 15 min. Thus, the observed $V_{{{\text{inc}}}}$ values using 808 nm light with Irgacure 819 is believed to originate from photochemical reactions that outweigh thermal reactions. A fundamentally important part of this study is to ensure that the size increase of the Au NPs is indeed due to the reduction of Ag ions on the surface of Au NPs. To verify this, energy dispersive X-ray spectroscopy (EDS) was employed to analyze the Au NPs after irradiation at 808 nm. The EDS spectrum (Fig. 4d) clearly shows the presence of both Au (initially present at 2.121 keV (M X-ray peak) and 9.712 keV (Lα peak)) and Ag (induced during the experiment at 2.984 keV (Lα peak) and 3.15 keV (Lβ2 peak)).

**Figure 5:**
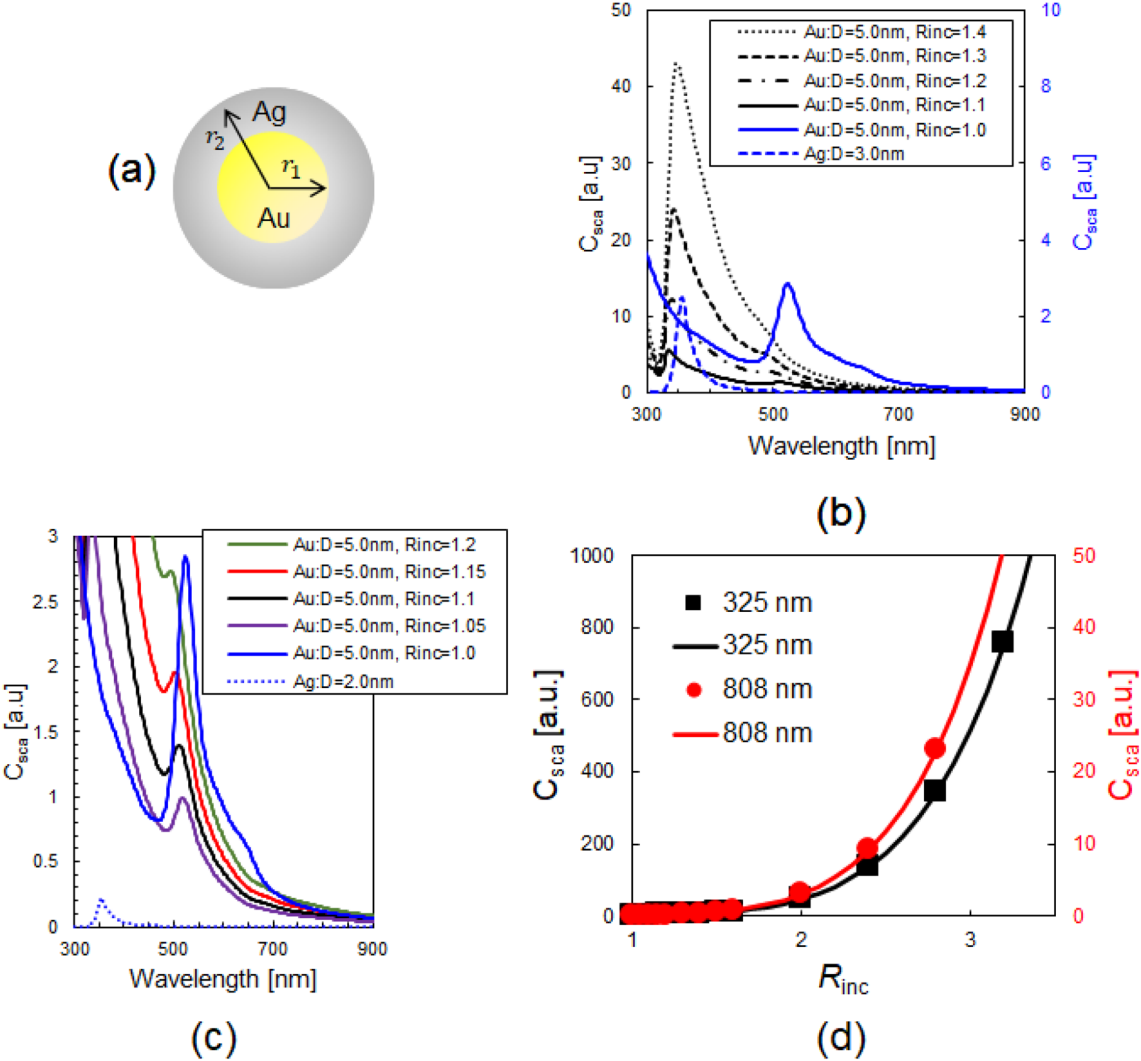
(**a**) Calculation model illustration of Ag coated Au NPs. $r_{1}$: radius of Au core, $r_{2}$: radius of core–shell structure. $r_{2}$ − $r_{1}$: Ag thickness. (**b**) and (**c**) Calculated values of $C_{sca}$ for different $R_{{{\text{inc}}}} \left( { = r_{2} /r_{1} } \right)$. Black curves in (**b**) correspond to the vertical axis on the left side and blue curves in (**b**) are associated with the vertical axis on the right side. (**c**) Detailed spectra with $R_{{{\text{inc}}}} < 1.2$. (**d**) $C_{sca}$ values obtained at 325 nm (black solid squares) and 808 nm (red solid circles). Fitting curves: $C_{sca} = a R_{inc}^{b}$ for 325 nm (black solid curve) and $C_{sca} = a R_{inc}^{b}$ for 808 nm (red solid curve).

Although direct characterization was not carried out for Ag deposition on Au NPs, the diameter increase suggested that the obtained nanostructures have Au–Ag core–shell structures. Based on this assumption, to investigate the field enhancement of metallic particles in Ag deposition, the scattering cross-section from the Ag coated Au NPs was calculated (see the calculation model illustrated in Fig. 5a). The polarizability of the ideal core–shell nanoparticle ($\alpha$) is given by^20^:

4 $$\alpha = 4\pi r_{2}^{3} \left[ {\frac{{\left( {\varepsilon_{1} + \varepsilon_{2} } \right)\left( {\varepsilon_{2} - \varepsilon_{3} } \right) + \left( {r_{1} /r_{2} } \right)^{3} \left( {\varepsilon_{1} - \varepsilon_{2} } \right)\left( {2\varepsilon_{2} + \varepsilon_{3} } \right)}}{{\left( {\varepsilon_{1} + 2\varepsilon_{2} } \right)\left( {\varepsilon_{2} + 2\varepsilon_{3} } \right) + 2\left( {r_{1} /r_{2} } \right)^{3} \left( {\varepsilon_{1} - \varepsilon_{2} } \right)\left( {\varepsilon_{2} - \varepsilon_{3} } \right)}}} \right]$$

where $r_{1}$ $= 2.5$ nm (the radius of the Au core) and $r_{2}$ is the radius of the Ag shell; and $\varepsilon_{1}$, $\varepsilon_{2}$, and $\varepsilon_{3}$ are the permittivity of the Au core, Ag shell, and water, respectively^21^. The scattering cross-section ($C_{sca}$) is given by the point dipole model^22^:

5 $$C_{sca} = \frac{{k^{4} }}{6\pi }\left| {\alpha^{2} } \right| = \frac{1}{6\pi }\left( {\frac{2\pi }{\lambda }} \right)^{4} \left| {\alpha^{2} } \right|$$

By substituting Eq. (4) into Eq. (5), $C_{sca}$ could be calculated. Figure 5b, c show the calculated $C_{sca}$, in which the plasmon resonance peaks of Au (approximately 520 nm^23^) and Ag (approximately 345 nm^24^) can be identified (please refer to section S2 in the Supplementary Information for detailed evaluation of the peak wavelength). With increasing $R_{{{\text{inc}}}} \left( { = r_{2} /r_{1} } \right)$, the Au plasmon peak decreased and the Ag plasmon peak becomes dominant in the $C_{sca}$ spectra. These numerical results show good agreement with the reported experimental results^25^. From these results, the $C_{sca}$ values at 325 and 808 nm were then plotted (Fig. 5d). From the fitting curves for 325 nm (black solid curve in Fig. 5d) and 808 nm (red solid curve in Fig. 5d) and their fitting parameters shown in Table 2, the $C_{sca}$ values were determined to be proportional to $R_{{{\text{inc}}}}^{6}$. This is reasonable because by substituting Eq. (4) into Eq. (5), $C_{sca}$ is proportional to $\left| {r^{6} } \right|$. From these results, the plasmonic field enhancement of metallic particles was then evaluated. By substituting the fitting curve in Fig. 3c of $R_{{{\text{inc}}}} = 0.0047 \times I^{0.75358} + 1$ into $C_{sca} = R_{{{\text{inc}}}}^{6}$, $C_{sca} \left( {R_{{{\text{inc}}}} } \right) = \left( {0.0047 \times I^{0.75358} + 1} \right)^{6}$ was obtained (black dashed curve in Fig. 3d). By comparing the experimental results (solid red squares in Fig. 3d) with calculated $C_{sca}$ (black dashed curve in Fig. 3d) for 808 nm light irradiation, the Ag deposition rate was far below the plasmonic field enhancement effect (black dashed curve in Fig. 3d). Therefore, we concluded that the observed Ag deposition by illumination with 808 nm light originated from the NF-assisted SHG (Fig. 1c) and subsequent excitation of $n - \pi^{*}$ transitions for the photocleavage of C–P bonds. Note that NF-assisted SHG does not yield the same results as when using a high-power laser (~ 10^9^ W/cm^2^)^26^, because a continuous laser with low power density (~ 1 W/cm^2^) was employed. In addition, because the wavelength of 808 nm for the NF-assisted process was far from the plasmon resonance wavelengths of 520 nm (Au) and 345 nm (Ag), the plasmon-induced SHG process^27^ can be excluded.

**Table 2:** Fitting parameters.

	*a*	SE	*b*	SE	COD
$C_{sca} = a R_{inc}^{b}$ black solid curve in Fig. 5d	0.77	0.02	5.92	0.02	0.999
$C_{sca} = a R_{inc}^{b} { }$ red solid curve in Fig. 5d	0.05	2.66 $\times$ 10^–4^	6.00	4.91 $\times$ 10^–3^	1.000

*SE* standard error, *COD* coefficient of determination.

## Conclusions

By introducing the NF source of Au NPs, Ag deposition to coat Au NPs was possible using a longer wavelength than the absorption edge wavelength of the photosensitive agent. By using TEM to evaluate the NP size, we revealed that the Ag shell growth rate followed from the squared power dependence, indicating that the Ag shell synthesis utilized two photons. The growth rate was much lower than that predicted from a plasmonic field enhancement effect, thus indicating that the observed Ag deposition originates from NF-assisted SHG and subsequent excitation. Future investigations will examine the exact role of the NF parameters, (e.g., size, structure, material, and polarization^28^) to provide a clearer understanding of how to control, and ultimately use, them for precise fabrication at the angstrom scale. In the future, we will conduct further investigations of the inner structure of the NPs using high-resolution TEM to better describe the structure of the core–shell nanostructures. The metallic nanoalloys composed of different materials that include core–shell structures are expected to exhibit different (and often better) behaviors than that of monometallic particles^29^. In particular, they present interesting optical properties^30,31^ that are very useful in many catalysis applications including catalytic converters in automobiles and electrochemical fuel cells. Because the near-field-assisted process has a low deposition rate, this technique is expected to improve the size control of the nanoalloys and the resultant property control.

## Material and methods

### Near field assisted silver synthesis on gold nanoparticles

As the ONF source, we used Au NPs (5 nm diameter, TANAKA Precious Metals). To deposit the Ag on the Au NPs, the latter were immersed in a photosensitive Ag ion aqueous solution containing 0.1 wt% AgNO_3_ and 0.2 wt% Irgacure 819 (bis-(2,4,6-trimethylbenzoyl)-phenylphosphine oxide, Aldrich), which is a Norrish type I free radical initiator with a quantum yield close to unity for the photocleavage of C–P bonds^17^.

### TEM evaluation and light irradiation experiments

We evaluated the size of the Au NPs before and after light irradiation using the following procedure:

1. We deposited Au NPs on the TEM carbon film (Product No. 6512, Nisshin EM Co. Ltd.). The solution containing the Au nanoparticles (0.004–0.005 wt% with pH = 4.5) was dispersed on the carbon film and the solvent was evaporated by heating at 75 °C using a hot plate for 1 min.
2. We evaluated the diameter of the Au NPs on the carbon film using TEM (JEM-2010F, JEOL Ltd.).
3. We performed light irradiation using a 325 nm laser as the FF light source and an 808 nm laser as the NF light source. To eliminate the effect of silver synthesis by the room light, the small beaker containing the Irgacure 819 was covered with aluminum foil before and after irradiation; moreover, all the experiments were performed under dark conditions with the room lights turned off. The light was irradiated onto the small beaker which contained all solutions, including AgNO_3_, Irgacure 819, and the TEM carbon film with the Au NPs. The light beam was irradiated perpendicular to the sample to avoid light absorption in the glass beaker. After irradiation, the carbon film was picked up from the beaker and set aside in the dark for more than 12 h to dry.
4. We evaluated the diameter of the NPs on the carbon film using TEM.

## Supplementary Information

Supplementary Information 1.

## Data Availability

Data that supports the findings of this study are available from the corresponding author upon reasonable request.
