# Supplementary material for: Synthesis of silver nanostructure on gold nanoparticle using near field assisted second harmonic generation
Source: Sci Rep. 2021 Mar 11;11:5642. doi: 10.1038/s41598-021-84944-w (PMC7970990; doi:10.1038/s41598-021-84944-w)
Supplement: Supplementary file 1 — Supplementary Information 1. [file 41598_2021_84944_MOESM1_ESM.docx]

Supplementary Information

Synthesis of silver nanostructure on gold nanoparticle using near field assisted second harmonic generation

Takashi Yatsui^1,*^, Felix Brandenburg^2^, Benjamin Leuschel^3,4^, and Olivier Soppera^3,4^

^1^Toyohashi University of Technology, 1-1 Hibarigaoka, Tenpaku-cho, Toyohashi, Aichi 441-8580, Japan

^2^The University of Tokyo, 7-3-1 Hongo, Bunkyo-ku, Tokyo 113-8656, Japan

^3^ Université de Haute-Alsace, CNRS, IS2M UMR 7361, F-68100 Mulhouse, France

^4^ Université de Strasbourg, France

### S1. NP size analysis

We evaluated the NP size using the software package NI vision (NATIONAL INSTRUMENTS Corp.). To evaluate the size of the NP smoothly, the raw data were edited manually to reduce any background noise prior to using NI vision. This process was conducted using Photoshop (Adobe Inc.). During the process, we included the scale bar from the original picture (with the original ratio) for accurate size determination of the NPs (see Fig. S1). We then evaluated the picture using NI vision. We used the calibration function in NI vision so that the scale bar was set to 50 nm. Next, we created binary data using the function of binary image inversion. We could then obtain the particle size using the particle analysis function.


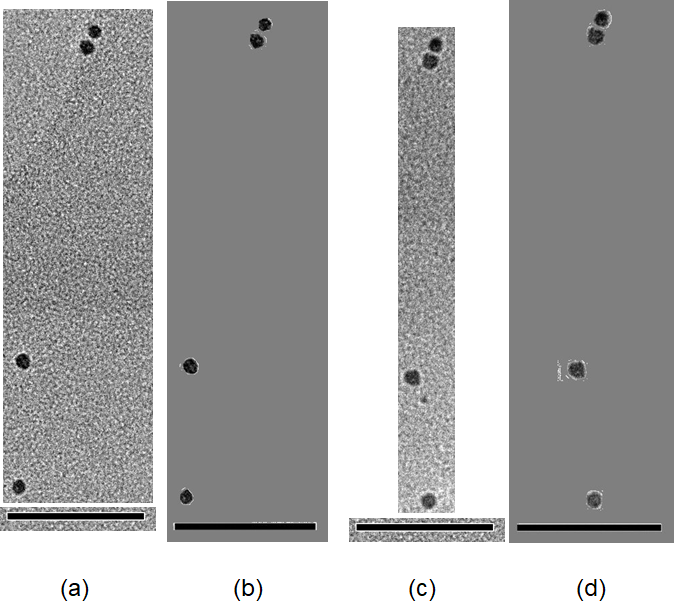


Figure S1: TEM images without photosensitive agent. (a) Before irradiation, (b) background-removed image of (a), (c) after 808 nm irradiation (20 mW for 15 min), and (d) background-removed image of (c). All scale bars are 50 nm.

### S2. Peak wavelength evaluations in the scattering cross-section

From the calculated values of $C_{sca}$ for the different $R_{\mathrm{inc}} \left( =r_{2}/r_{1} \right)$ shown in Figs. 5(b) and 5(c), we plotted the wavelengths of the two main peaks in Figs. S2(a) and S2(b). As shown in Fig. S2(a), the longer peak wavelength increased as $R_{\mathrm{inc}}$ decreased, and reached the peak wavelength (528 nm) of Au NP alone (blue solid curve in Fig. 5(b)). Therefore, this peak is considered to be the plasmon resonance peak of Au NP. This value corresponds to the reported value (approximately 520 nm)^S1^. Moreover, as shown in Fig. S2(b), the shorter peak wavelength increased as $R_{\mathrm{inc}}$ increased, and reached the peak wavelength (358 nm) of Ag NP alone (blue dashed curve in Fig. 5(b)). Therefore, this peak is considered to be the plasmon resonance peak of Ag NP. This value corresponds to the reported value (approximately 345 nm)^S2^.


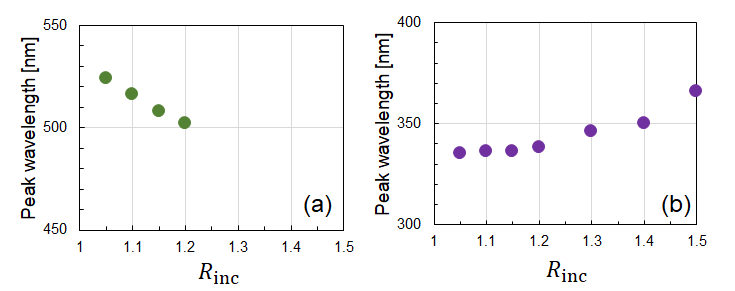


Figure S2: Peak wavelength as a function of $R_{\mathrm{inc}}$ obtained from Fig. 5(b) and 5(c). (a) Longer and (b) shorter wavelength peak.

### S3. Absorption spectra of Irgacure 819

### After acquiring the reference spectra of the solvent of isopropyl alcohol, we obtained the absorption spectra of Irgacure 819 (black dots in Figs. S3(a) and S3(b)) in which we set the zero at the wavelength of 850 nm. From the raw data of the absorbance, we fitted this with the weighted moving average (Savitzky–Golay)^S3^ using 50 data points (red solid curves in Figs. S3(a) and S3(b)). The absorbances of Irgacure 819 were 2.62$\times{10}^{-4}$ and 3.35 for 808 nm and 404 nm, respectively.

### References


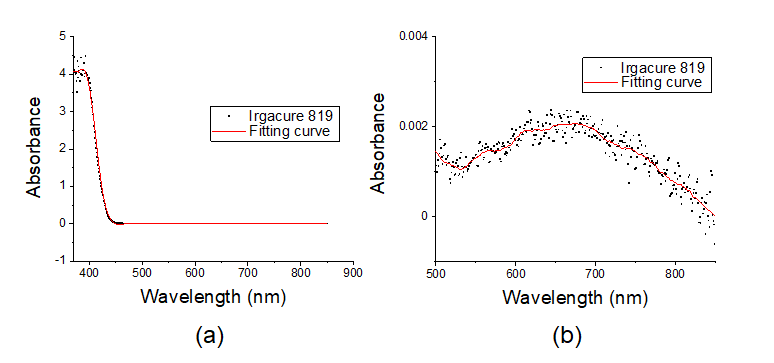
Figure S3: Absorption spectra of Irgacure 819. Spectral range for (a) $350 <\lambda<850$ nm and (b) $500 <\lambda<850$ nm.

### [s1] Yatsui, T. et al. Enhanced photo-sensitivity in a Si photodetector using a near-field assisted excitation. Communications Physics **2**, 62 (2019).

### [s2] Wilson, O., Wilson, G. J. & Mulvaney, P. Laser Writing in Polarized Silver Nanorod Films. *Advanced Materials* **14**, 1000-1004 (2002).

### [s3] Savitzky, A. & Golay, M. J. E. Smoothing and Differentiation of Data by Simplified Least Squares Procedures. *Anal. Chem.* **8**, 1627–1639 (1964).
